# Estimating the loss of lifetime function using flexible parametric relative survival models

**DOI:** 10.1186/s12874-019-0661-8

**Published:** 2019-01-28

**Authors:** Lasse H. Jakobsen, Therese M.-L. Andersson, Jorne L. Biccler, Tarec C. El-Galaly, Martin Bøgsted

**Affiliations:** 1 Department of Clinical Medicine, Aalborg University, Sdr. Skovvej 15, Aalborg, 9000 Denmark; 2 Department of Hematology, Aalborg University Hospital, Sdr. Skovvej 15, Aalborg, 9000 Denmark; 3 Department of Medical Epidemiology and Biostatistics, Karolinska Institutet, Nobels Väg, Stockholm, 171 65 Sweden

**Keywords:** Loss of lifetime, Relative survival, Extrapolation, Cancer survival

## Abstract

**Background:**

Within cancer care, dynamic evaluations of the loss in expectation of life provides useful information to patients as well as physicians. The loss of lifetime function yields the conditional loss in expectation of life given survival up to a specific time point. Due to the inevitable censoring in time-to-event data, loss of lifetime estimation requires extrapolation of both the patient and general population survival function. In this context, the accuracy of different extrapolation approaches has not previously been evaluated.

**Methods:**

The loss of lifetime function was computed by decomposing the all-cause survival function using the relative and general population survival function. To allow extrapolation, the relative survival function was fitted using existing parametric relative survival models. In addition, we introduced a novel mixture cure model suitable for extrapolation. The accuracy of the estimated loss of lifetime function using various extrapolation approaches was assessed in a simulation study and by data from the Danish Cancer Registry where complete follow-up was available. In addition, we illustrated the proposed methodology by analyzing recent data from the Danish Lymphoma Registry.

**Results:**

No uniformly superior extrapolation method was found, but flexible parametric mixture cure models and flexible parametric relative survival models seemed to be suitable in various scenarios.

**Conclusion:**

Using extrapolation to estimate the loss of lifetime function requires careful consideration of the relative survival function outside the available follow-up period. We propose extensive sensitivity analyses when estimating the loss of lifetime function.

**Electronic supplementary material:**

The online version of this article (10.1186/s12874-019-0661-8) contains supplementary material, which is available to authorized users.

## Background

Dynamic survival prediction is important in cancer care, where prognostic assessments are given numerous times during diagnosis, treatment, and post-treatment follow-up. A popular measure for characterizing the severity of a disease is the expected amount of lifetime lost due to the disease as compared to the general population. This measure is known as the *loss in expectation of life* and may be computed as the difference between the area under the general population and patient survival curves [1]. The loss in expectation of life has previously been used to characterize the disease burden within colon cancer and acute myeloid leukemia [2, 3]. The *loss of lifetime* function generalizes this measure by dynamically evaluating the loss in expectation of life, yielding the conditional number of years lost due to cancer given survival up to specific time points.

Due to the occurrence of censoring, computing the loss of lifetime function typically requires extrapolation of both the patient and general population survival function. Generally, extrapolation of survival functions estimated from censored time-to-event data is challenging since there is no way to evaluate the extrapolation accuracy and even a well-fitted model may extrapolate poorly. Nonetheless, in order to provide estimates of the long-term effects of a given treatment, extrapolated survival probabilities are often required in the analysis of data from clinical trials [4].

An extensive literature exists on techniques for extrapolating survival functions. Jackson et al. reviewed methods for incorporating external data, such as register data or national life tables, to extrapolate survival functions [5]. Such approaches require a quantification of how the survival in the present patient population and the external data differ and assumptions about how this will continue beyond the follow-up. In particular, extrapolation through the relative survival function has been proposed for both grouped and individual-level data, which has demonstrated improved accuracy in comparison to models for the all-cause survival function [1, 6]. Andersson et al. examined the accuracy of the loss in expectation of life estimates calculated by three types of relative survival models [1]. However, none of these assessments were conducted for the entire loss of lifetime function.

In the following article, we compute the loss of lifetime function using previously introduced extrapolation approaches. In addition, a new flexible parametric relative survival model based on mixture cure models and spline-based proportional hazards models is introduced [7, 8]. We expand the study of Andersson et al. [1] by evaluating the accuracy of the entire loss of lifetime function based on various extrapolation approaches in a simulation study and in data from the Danish Cancer Registry where complete follow-up was available. In addition, as a clinically motivated example, the loss of lifetime function is computed for three lymphoma types using recent data from the Danish Lymphoma Registry.

## Methods

### Relative survival

The relative survival function is commonly used to describe the disease-specific (net) survival without requiring cause of death information. Given covariate vector ***z***, patient population (all-cause) survival function *S*(*t*|***z***), and general population survival function, *S*^∗^(*t*|***z***), the relative survival function is given by

1 $$ R(t|\boldsymbol{z}) = \frac{S(t|\boldsymbol{z})}{S^{*}(t|\boldsymbol{z})}.  $$

By using the relation between the hazard function and the survival function, the all cause hazard function corresponding to *S*(*t*|***z***) can be written as

2 $$ h(t|\boldsymbol{z}) = h^{*}(t|\boldsymbol{z}) + \lambda(t|\boldsymbol{z}),  $$

where *h*^∗^(*t*|***z***) is the general population hazard function and *λ*(*t*|***z***) is termed the *excess hazard function* or *excess mortality*. Both *h*^∗^(*t*|***z***) and *S*^∗^(*t*|***z***) are usually computed from publicly available life tables matched on variables such as age, sex, and calendar year. The most popular way to include covariate effects is the proportional excess hazard model with a parametric specification of the baseline excess hazard [9, 10].

### Parametric cure models

In survival analysis, cure models are used to provide useful information, particularly in cancers where the patient hazard function reaches the same level as the general population hazard function after some time [7, 11]. This corresponds to the relative survival reaching a plateau and the patients still alive after this time point are considered statistically cured. The main parameter of interest in cure models is the proportion of patients reaching statistical cure, also known as the cure proportion. Cure models are commonly divided into mixture and non-mixture cure models [7]. In mixture cure models, the patient population is considered a mixture of cured and uncured individuals. The relative survival is a mixture of a relative survival function for the cured and uncured patients, i.e.,

3 $$ R(t|\boldsymbol{z}) = \frac{S(t|\boldsymbol{z})}{S^{*}(t|\boldsymbol{z})} = \pi(\boldsymbol{z}) + [1 - \pi(\boldsymbol{z})] S_{u}(t|\boldsymbol{z}),   $$

where *π*(***z***) is the, potentially covariate dependent, cure proportion and *S*_*u*_(*t*|***z***) is the relative survival function of the uncured patients. The cure proportion can be modelled through a link function, e.g., with a logistic, identity, or log-log link function [7]. The function *S*_*u*_(*t*|***z***) can conveniently be modelled by regular parametric survival models, such as a Weibull model, a log-normal model, or more flexible alternatives such as a Weibull-Weibull mixture model [12]. The model is estimated by maximum likelihood where the only external information needed is the general population hazard at the observed event times (see Lambert et al. [7] for the likelihood function).

Non-mixture cure models are of a less intuitive form:

4 $$ R(t|\boldsymbol{z}) = \frac{S(t|\boldsymbol{z})}{S^{*}(t|\boldsymbol{z})} = \pi(\boldsymbol{z})^{1 - \widetilde{S}(t|\boldsymbol{z})},  $$

where the function $\widetilde {S}(t|\boldsymbol {z})$ is a proper survival function which does not have an intuitive interpretation like *S*_*u*_(*t*|***z***). By rewriting the non-mixture cure model, it can be formulated as a mixture cure model, with $\left (\pi (\boldsymbol {z})^{1-\widetilde {S}(t|\boldsymbol {z})} - \pi (\boldsymbol {z})\right)/(1 - \pi (\boldsymbol {z}))$ as the relative survival function of the uncured patients [7]. Thus, estimation of the non-mixture cure model can be carried out similarly to that of mixture cure models.

### Flexible parametric cure models

Royston and Parmar introduced a flexible parametric proportional hazards model by using restricted cubic splines to model the baseline hazard function (on the log cumulative hazard scale) [8]. This approach was applied to relative survival by Nelson et al. where the log-cumulative excess hazard was modelled by restricted cubic splines [10]. Including covariate effects, the relative survival by Nelson et al. is given by

5 $$ \log(-\log(R(t| \boldsymbol{z})) = s_{0}(x;\boldsymbol{\gamma}_{0}) + \boldsymbol{z}^{T} \boldsymbol{\beta} + \sum\limits_{i = 1}^{p} s_{i}(x;\boldsymbol{\gamma}_{i})z_{i},  $$

where *x*= log(*t*), *p* is the number of time-varying covariate effects, *s*_0_(*x*;***γ***_0_) is a baseline restricted cubic spline, ***β*** is a vector of regression coefficients, and *s*_*i*_(*x*;***γ***_*i*_) is a spline corresponding to the *i*^th^ covariate, providing a time varying coefficient. For the *i*^th^ spline, *K*_*i*_ knots, $\phantom {\dot {i}\!}k_{i1}< k_{i2}<...< k_{iK_{i}}$, are selected on the log-time scale. The spline is then given as a linear combination of base functions defined through the chosen knots, i.e., $s_{i}(x;\boldsymbol {\gamma }_{i}) = {\sum \nolimits }_{j = 0}^{K_{i} - 1} v_{ij}(x)\gamma _{ij}$, where ***γ***_*i*_ are model parameters. The base functions are given by *v*_*i*0_(*x*)=1, *v*_*i*1_(*x*)=*x*, and

6 $$ v_{ij}(x) = (x - k_{ij})^{3}_{+} - \lambda_{ij}(x - k_{i1})^{3}_{+} - (1 - \lambda_{ij}) (x - k_{iK_{i}})^{3}_{+},  $$

for *j*=2,...,*K*_*i*_−1, where $\lambda _{ij} = \frac {k_{iK_{i}} - k_{ij}}{k_{iK_{i}} - k_{i1}}$ and *x*_+_= max(*x*,0). Generally, the number and placement of the knots in the different spline functions do not need to be the same.

Andersson et al. used (5) to establish a flexible parametric cure model [13]. This model is formulated similarly to (5), but the basis functions of the splines are adjusted to ensure that the relative survival has zero slope after a preselected time point which is used as the last knot in all spline functions, i.e., $k_{K} = k_{0K_{0}} = k_{1K_{1}} = \cdots = k_{pK_{p}}\phantom {\dot {i}\!}$. The cure proportion is then estimated by *R*(*k*_*K*_). Rewriting (5) we obtain

7 $$ \begin{aligned} &R(t|\boldsymbol{z}) = \exp\\& \left(\,-\,\exp\!\left(\gamma_{00} + \boldsymbol{z}^{T} \boldsymbol{\beta}\right)\!\exp\!\left({\sum\limits_{i = 1}^{K_{0} - 1} v_{i}(x)\gamma_{i} + \sum\limits_{i = 1}^{p} s_{i}(x;\boldsymbol\gamma_{i})z_{i}}\right)\right)\!. \end{aligned}  $$

Hence, the model by Andersson et al. can be viewed as a non-mixture cure model where the cure proportion is modelled through the baseline spline parameter, *γ*_00_, and the fixed covariate effects, ***z***^*T*^***β***, while the remaining parameters are used to model $1 - \widetilde S(t)$ [13]. While this model provides a flexible framework for estimating the cure proportion in cancer studies, the assumption of statistical cure after the last knot is strong. Therefore, we introduce a new flexible parametric cure model which combines regular mixture cure models with flexible parametric survival models. The model is specified by (3) with

8 $$ S_{u}(t|\boldsymbol{z}) \,=\, \exp\!\left(\!-\exp\left({s_{0}(x;\boldsymbol\gamma_{0}) + \boldsymbol{z}^{T}\boldsymbol\beta + \sum\limits_{i = 1}^{p} s_{i}(x;\boldsymbol\gamma_{i})z_{i}}\right)\right).   $$

Similarly to the more simple cure models presented in Lambert et al. [7], *π*(***z***) can be modelled by various link functions and the relative survival cannot fall below *π*(***z***), thus ensuring statistical cure. The model is fitted by maximum likelihood using the likelihood of the mixture cure model. This cure model enables flexible modelling of the relative survival without the strong assumption of cure after the last knot while providing the more intuitive interpretation of a mixture cure model. Additionally, in this model, the modelling of the cure proportion becomes more clearly separated from the modelling of *S*_*u*_(*t*).

### The loss of lifetime function

The conditional expected residual lifetime given survival until a time point *t* for individuals with covariate vector ***z*** can be computed by $\int \limits _{t}^{\infty } S(u|\boldsymbol {z})du / S(t|\boldsymbol {z})$. Based on this property, the loss of lifetime function can be computed by

9 $$ L(t|\boldsymbol{z}) = \frac{\int_{t}^{\infty} S^{*}(u|\boldsymbol{z})du}{S^{*}(t|\boldsymbol{z})} - \frac{\int_{t}^{\infty} S(u|\boldsymbol{z})du}{S(t|\boldsymbol{z})},   $$

which is the difference in expected residual lifetime after time point *t* between the general population and the patients.

Extrapolation of both *S*^∗^(·|***z***) and *S*(·|***z***) is required to compute (9) since the survival distributions typically cannot be fully estimated due to censoring. Similarly to Andersson et al. [1], the extrapolation of the expected survival, *S*^∗^(·), can be accomplished by using the method of Ederer et al. [14] (Ederer I) and by making assumptions about the future population mortality rates. The latter can be carried out by using mortality rates from the last available time point or, if available, by using predicted future mortality rates.

For the patient survival, we apply the relative survival factorization, i.e., *S*(*t*)=*S*^∗^(*t*)*R*(*t*), such that the extrapolation is based on the relative survival and the general population survival. Extrapolation of *R*(·) can be enabled by fitting a parametric relative survival model [1]. Since some cancer patient groups experience statistical cure after some time while others experience persistent excess mortality, several assumptions on the relative survival can be applied. We consider three flexible parametric relative survival models, which mainly differ in the tail:

- the Nelson et al. [10] relative survival (NRS) model, which is linear on the log cumulative excess hazard scale after the last knot,
- the Andersson et al. [13] relative survival (ARS) model, which is constant on the log cumulative excess hazard scale after the last knot and thereby incorporates statistical cure, and
- the flexible mixture cure (FMC) model in (8), which incorporates statistical cure, but is not restricted to a constant log cumulative excess hazard after the last knot.

Due to their flexibility, the three models typically behave similarly within the first part of the follow-up, but may produce different survival trajectories beyond the available follow-up. In cure models, the relative survival cannot fall below *π*, and thus these models have a parameter to control the asymptote of the relative survival. Therefore, in cases where statistical cure occurs, cure models may improve extrapolation as compared to non-cure models. In cases where statistical cure does not occur, cure models may provide too optimistic extrapolations and hence may not be appropriate. However, in such cases, the introduced FMC model is expected to estimate *π* close to zero such that the fit is mainly based on the flexible survival function, *S*_*u*_(*t*). In the ARS model, letting *π*=0, substantially affects the survival function since this forces *R*(*k*_*K*_)=0. Therefore, we consider the FMC model a hybrid between the NRS and ARS models.

### Implementation

Initial values for the optimization procedure for the FMC model were chosen by first fitting a Weibull parametric cure model using only fixed covariate effects, i.e., fitting model (3) with a Weibull formulation of *S*_*u*_(*t*) and a logistic link for *π*. For the cure proportion, initial values were found by fitting a linear model with the predicted cure proportions scaled by the chosen link function as response and the cure proportion covariates as explanatory variables. For the relative survival of the uncured, initial values were found by fitting a linear model with the log-log transformed predicted relative survival of the uncured at the observed event times as response and the splines and covariates of *S*_*u*_(*t*) as explanatory variables. The splines do not guarantee that *S*_*u*_(*t*) is proper, but this can be obtained by adding a penalty for negative values of *h*_*u*_(*t*)=−*d*/*dt* log*S*_*u*_(*t*) similarly to Liu et al. [15]. In particular, the term

10 $$ \frac{\kappa}{2} \sum\limits_{j = 1}^{n} h_{u}(t_{j}|\boldsymbol{z}_{j})^{2}\mathbf{1}\left[h_{u}(t_{j}|\boldsymbol{z}_{j}) < 0\right]  $$

is subtracted from the log-likelihood, where *t*_*j*_ and ***z***_*j*_ are the observed follow-up time and covariate vector, respectively, of patient *j*. Initially, *κ* is 1, but doubles until no negative values of *h*_*u*_ are obtained. Orthogonalization of the base functions of the restricted cubic splines has previously been recommended due to the potential correlation between the base functions [16]. We employed a QR-decomposition approach to carry out the orthogonalization.

Choosing the number and location of the knots is a key issue in spline-based models. Similarly to Royston and Parmar, the knots of the FMC model were selected according to the quantiles of the uncensored event times [8]. In a simulation study, Rutherford et al. [16] concluded that complex hazard shapes can adequately be captured by the spline-based model of Royston and Parmar [8] provided that a sufficient number of knots are selected. In particular, the survival model was rather insensitive to the number of knots and it was argued that the results should also be valid in relative survival and cure models [16].

All analyses were performed in the statistical programming language R. For the purpose of this article, the NRS and ARS models were fitted using the package rstpm2 [17]. Functions for estimating the presented FMC model and computing the loss of lifetime function were assembled in the R-package cuRe (see https://github.com/LasseHjort/cuRe). The package also enables estimation of the expected residual lifetime, restricted expected residual lifetime, and restricted loss of lifetime using any of the models considered here. The integrals of the loss of lifetime function were computed numerically by Gauss-Legendre quadrature, while the point-wise variance of the loss of lifetime function was estimated using the delta method and numerical differentiation.

## Results

### Simulation study

#### Simulation design

We simulated data according to selected relative survival scenarios by assuming independence between the relative survival and general population survival times. Similarly to Rutherford et al. [18], we used the following simulation scheme:

- Draw a general population survival time *T*_*E*_ from *S*^∗^.
- Draw a relative survival time, *T*_*R*_ from *R*.
- Draw a censoring time *T*_*C*_ from *C*.
- The observed follow-up time is given by *T*= min(*T*_*R*_,*T*_*E*_,*T*_*C*_) and the event indicator is *δ*=***1***[min(*T*_*R*_,*T*_*E*_)≤*T*_*C*_].

**Fig. 1:**
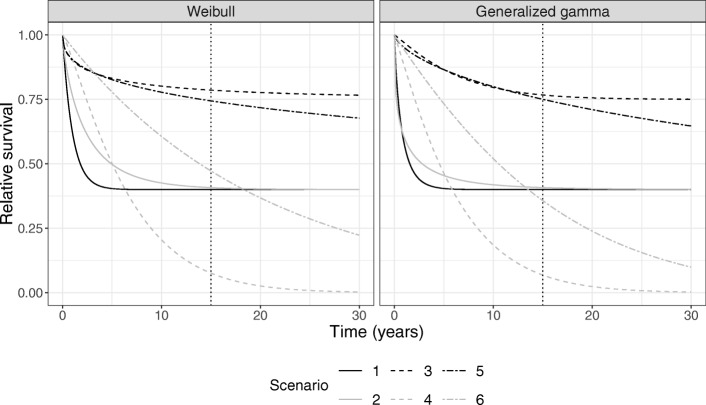
Relative survival functions used to simulate net survival times. In scenario 1, 2, and 3, follow-up times were simulated from a Weibull (generalized gamma) cure model with varying cure proportions, and in scenario 4, 5, and 6, the follow-up times were simulated from a Weibull (generalized gamma) relative survival model

The general population survival distribution, *S*^∗^(*t*), was chosen corresponding to 50-, 60-, and 70-year-old female patients diagnosed in 1980. For this purpose, we used the Danish general population mortality rates published by the Human Mortality Database [19]. The relative survival, *R*(*t*), was determined by a Weibull mixture cure model according to the scenarios in Fig. 1. In scenario 1, 2, and 3, the cure proportion was 40%, 40%, and 75% and cure occurred within the available follow-up, just outside the follow-up, and many years after the last follow-up time, respectively. In scenarios 4, 5, and 6, the cure proportion was zero and therefore the relative survival function corresponded to a regular Weibull model. Scenario 5 was similar to 3 within the follow-up, but differed beyond the follow-up. In scenario 4, most patients died within the follow-up and scenario 6 was included as an example of a clear absence of cure within the follow-up. In scenarios where *R*(*t*) had a cure proportion, follow-up times were set to *∞*, if there was no solution to the equation *R*(*t*)=*U*, where *U* is uniformly distributed between 0 and 1. To examine the extrapolation performances under different trajectories, we repeated the simulations after replacing the Weibull distribution with the generalized gamma distribution.

To mimic typical register data, the censoring times were simulated from a uniform distribution, *C*, between 0 and 15 years. Using *S*^∗^ and *R*, the true loss of lifetime function was obtained by inserting into (9). All scenarios were simulated 500 times with a sample size of 1000.

For estimation of the loss of lifetime function, we considered five models (Table 1). In order to obtain the same number of parameters in each model, an additional knot was required for model B and C, which was placed late in the follow-up, while for model D and E the number of knots was decreased by one since these contain an explicit parameter for the cure proportion. Extrapolation using model A and B was considered by Andersson et al. [1]. We considered a special case of the latter model, where the last knot was placed beyond the available follow-up. We also considered two instances of the FMC model, i.e., D with conventional knot placement and E where the knots were placed in the beginning of the follow-up.

**Table 1:** Specification of models used to estimate the loss of lifetime function

Model	Model	Nr. knots	Knot locations
A	NRS	6	0%, 20%, 40%, 60%, 80%, and 100% quantiles of the uncensored event times.
B	ARS	7	0%, 20%, 40%, 60%, 80%, and 100% quantiles of the uncensored event times with an additional knot placed at 10 years.
C	ARS	7	0%, 20%, 40%, 60%, and 80% quantiles of the uncensored event times. The last knot is placed at 80 years and an additional knot is placed at 10 years.
D	FMC	5	0%, 25%, 50%, 75%, and 100% quantiles of the uncensored event times.
E	FMC	5	First uncensored event time, 0.5, 1, 2, and 5 years.

NRS: Nelson et al. [10] relative survival model, ARS: Andersson et al. [13] relative survival model, FMC: Flexible mixture cure model

For each model, the loss of lifetime function was computed and the bias was measured by $D(t) = \widehat {L}(t) - L(t)$. The integral, $\int _{0}^{15}|D(u)|du$, was used to measure the bias of the loss of lifetime estimate during the entire follow-up period.

#### Simulation results

**Fig. 2:**
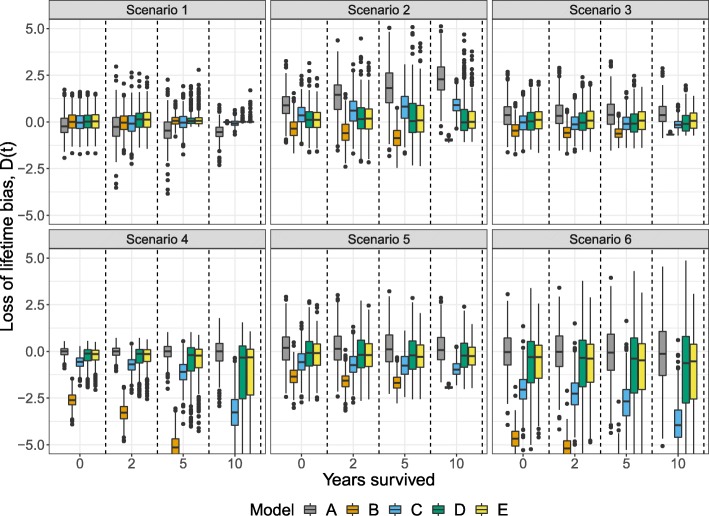
Loss of lifetime bias, *D*(*t*), of the models in Table 1 at time 0, 2, 5, and 10 years in 50-year-old patients following six Weibull relative survival scenarios

In scenarios with statistical cure (scenario 1, 2 and 3), all models had comparable performances at time zero for 50-year-old patients (Fig. 2). In scenarios 1 and 3, the bias was fairly low for all models at all time points, but in scenario 2, the non-cure model, A, yielded increasingly upward biased estimates. In scenarios without statistical cure (scenario 4, 5, and 6), the diversity between the models became larger. In these scenarios, the non-mixture cure models, B and C, underestimated the loss of lifetime, most markedly seen in model B which assumes cure within the follow-up period.

Generally, the FMC models, D and E, showed good performance both in scenarios with statistical cure occurring within and beyond the available follow-up. In scenarios where statistical cure did not occur, the performance of the FMC models was comparable to model A, but the biases were more dispersed for later time point, especially in scenario 4 and 6. At ten years, the biases of model E were slightly less dispersed compared to model D.

**Fig. 3:**
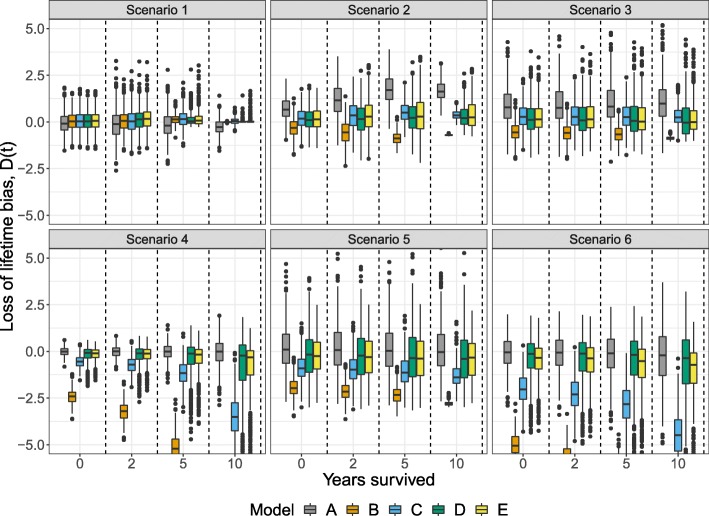
Loss of lifetime bias, *D*(*t*), of the models in Table 1 at time 0, 2, 5, and 10 years in 50-year-old patients following six generalized gamma relative survival scenarios

Table 2 shows the integrated loss of lifetime biases for 50-, 60-, and 70-year-old patients. In general, the integrated overall biases were consistent with Fig. 2 where model A, D, and E performed well across the six scenarios. In comparison to model D, model E was largely producing less biased estimates, while only being slightly worse than model A in scenario 4 and 6. Generally, the loss of lifetime bias decreased with increasing age and hence reduced the differences between the models. Despite the bias reduction in 70-year-olds, model B still resulted in a relatively large bias in scenario 4 and 6. The results were similar in the generalized gamma case (Fig. 3 and Table 3). In particular, the models A and E showed satisfactory performance in all scenarios while model D was more biased in scenario 6. Also in the generalized gamma case, model E had slightly lower integrated bias compared to model D in scenario 4, 5, and 6.

**Table 2:** The integrated loss of lifetime bias in the Weibull scenario, computed by integrating |*D*(*t*)| from 0 to 15 years

Age	Scenario	pi	Model A	Model B	Model C	Model D	Model E
50	1	0.40	8.6(0.9-39.2)	2.4(0.5-10.7)	4.1(0.9-19.5)	2.4(0.2-18.5)	2.6(0.3-28.6)
	2	0.40	28.9(5.5-66.9)	11.7(6.0-23.1)	12.7(3.7-35.3)	11.1(0.6-68.0)	9.3(0.7-52.1)
	3	0.75	8.9(0.7-42.7)	8.9(4.3-15.6)	4.8(0.9-16.4)	7.5(0.5-31.5)	7.2(0.2-23.9)
	4	0.00	6.5(0.3-34.5)	144.8(123.4-171.2)	37.2(8.9-82.1)	24.9(0.3-111.8)	18.1(0.2-104.6)
	5	0.00	11.0(0.2-43.3)	25.4(14.8-35.4)	13.0(3.2-31.0)	12.8(0.4-33.8)	9.6(0.3-30.9)
	6	0.00	18.1(0.5-65.4)	106.6(71.6-127.7)	49.2(7.9-92.4)	36.2(0.6-103.7)	23.9(0.2-89.4)
60	1	0.40	6.0(1.3-19.4)	1.9(0.4-8.0)	3.1(0.6-13.5)	2.1(0.2-14.7)	2.4(0.2-17.6)
	2	0.40	14.9(2.6-45.4)	6.4(3.4-14.9)	7.7(2.0-26.2)	7.7(0.6-40.2)	6.6(0.2-42.5)
	3	0.75	7.2(0.4-39.6)	4.2(1.9-10.0)	4.0(0.4-22.7)	5.4(0.3-28.2)	4.7(0.3-19.6)
	4	0.00	5.7(0.3-24.1)	79.5(64.6-93.2)	21.0(6.1-44.4)	14.2(0.3-62.4)	10.2(0.1-49.8)
	5	0.00	7.5(0.3-33.4)	10.7(5.1-18.1)	5.6(1.5-18.6)	7.2(0.5-26.0)	5.0(0.2-17.9)
	6	0.00	10.9(0.6-37.3)	48.2(36.4-61.2)	18.5(4.1-42.5)	16.8(1.2-50.9)	11.2(0.3-45.3)
70	1	0.40	3.6(0.9-12.4)	1.5(0.2-4.8)	2.2(0.4-7.3)	1.7(0.1-8.8)	2.0(0.1-12.9)
	2	0.40	6.2(1.2-20.8)	3.4(1.5-8.8)	4.0(0.9-14.2)	4.7(0.3-19.4)	4.3(0.3-19.2)
	3	0.75	4.9(0.3-20.6)	2.4(0.8-7.0)	3.3(0.3-12.9)	3.6(0.2-19.0)	2.8(0.2-11.2)
	4	0.00	4.3(0.3-16.1)	34.9(26.9-44.4)	9.6(3.5-23.3)	7.3(0.2-31.3)	5.7(0.1-27.5)
	5	0.00	5.3(0.4-25.2)	3.9(1.7-8.9)	3.5(0.6-14.8)	4.3(0.2-18.5)	2.9(0.1-10.5)
	6	0.00	6.0(0.3-21.7)	16.5(9.9-23.8)	6.2(1.8-16.6)	6.9(0.2-23.7)	5.1(0.2-17.4)

The loss of lifetime was computed for 50-, 60-, and 70-year-old patients. The mean and range from the 500 simulations are provided

**Table 3:** The integrated loss of lifetime bias in the generalized gamma scenario, computed by integrating |*D*(*t*)| from 0 to 15 years

Age	Scenario	pi	Model A	Model B	Model C	Model D	Model E
50	1	0.40	6.8(0.6-30.1)	2.4(0.4-11.3)	4.0(0.5-21.0)	2.6(0.3-29.8)	3.1(0.3-30.5)
	2	0.40	23.1(5.0-48.1)	10.2(5.7-18.3)	7.1(1.8-22.8)	8.5(0.6-39.3)	10.3(0.7-43.8)
	3	0.75	17.4(1.5-74.8)	10.2(4.2-19.8)	7.6(1.0-31.0)	11.2(0.8-63.1)	10.0(0.3-56.2)
	4	0.00	6.7(0.2-32.6)	146.5(121.6-166.8)	39.2(10.4-75.0)	22.8(0.2-123.1)	15.4(0.2-95.0)
	5	0.00	14.8(0.6-88.3)	35.9(22.7-45.6)	18.0(3.2-39.4)	18.4(0.6-77.5)	13.7(0.3-40.3)
	6	0.00	16.1(0.6-72.9)	130.5(108.5-153.0)	56.1(9.8-99.7)	36.4(0.5-117.8)	21.3(0.8-100.1)
60	1	0.40	4.7(0.9-16.5)	1.9(0.2-7.5)	3.0(0.3-12.1)	2.2(0.2-14.3)	2.8(0.1-21.5)
	2	0.40	12.0(2.7-35.4)	5.5(3.1-10.9)	4.6(1.0-19.4)	5.7(0.4-32.1)	7.2(0.5-35.0)
	3	0.75	11.1(1.0-53.0)	5.2(2.2-11.4)	6.1(0.7-31.7)	7.4(0.8-37.6)	7.5(0.4-31.0)
	4	0.00	5.8(0.2-22.2)	80.3(65.1-96.0)	21.8(7.3-42.4)	13.9(0.3-66.4)	9.5(0.2-49.4)
	5	0.00	10.2(0.7-55.2)	14.8(6.9-22.4)	7.4(1.6-29.4)	9.6(0.3-33.6)	6.8(0.2-22.0)
	6	0.00	9.8(0.3-37.7)	62.2(44.7-77.0)	24.1(5.9-51.5)	17.1(0.7-64.5)	11.4(0.6-54.9)
70	1	0.40	3.1(0.6-11.0)	1.4(0.2-4.8)	2.2(0.2-7.4)	1.8(0.2-8.8)	2.3(0.1-12.7)
	2	0.40	5.5(1.0-16.7)	2.6(1.4-6.0)	2.9(0.4-11.1)	3.2(0.2-13.6)	4.2(0.3-14.6)
	3	0.75	6.4(0.4-30.7)	2.8(1.0-8.8)	4.1(0.3-20.0)	4.6(0.4-24.6)	4.5(0.4-17.6)
	4	0.00	4.2(0.3-14.9)	35.4(26.7-43.6)	10.2(3.9-23.9)	7.4(0.3-30.9)	5.4(0.2-24.0)
	5	0.00	6.1(0.2-26.8)	4.9(2.3-10.5)	3.9(0.6-17.2)	4.8(0.4-26.0)	3.6(0.1-13.1)
	6	0.00	6.1(0.4-23.2)	22.4(14.5-30.4)	8.0(2.4-22.7)	7.2(0.7-29.7)	5.2(0.4-25.1)

The loss of lifetime was simulated for 50-, 60-, and 70-year-old patients. The mean and range from the 500 simulations are provided

### Analysis of Danish cancer registry data

#### Data description

To investigate the performance of the models in Table 1 in cancer survival data, we analyzed data from the Danish Cancer Registry [20] on patients with colon cancer (*n*=4558), breast cancer (*n*=21,731), bladder cancer (*n*=11,738) and malignant melanoma (*n*=2404). To achieve (almost) complete follow-up, we included patients diagnosed in the period 1960–1975, who were older than 50 years at diagnosis. The diseases were chosen based on the same considerations as in Andersson et al. [1], i.e., colon cancer typically displays statistical cure, bladder cancer a constant excess hazard, melanoma a rather high survival rate, and breast cancer is seen in both young and old patients. Patients were followed until the end of 2016, where alive patients were censored and follow-up was measured from diagnosis until death or censoring. For the purpose of investigating the extrapolation performance, we restricted the follow-up to 16 years by censoring patients alive in January 1976 and divided patients into age groups; 50–59, 60–69, 70–79, 80+. The true loss of lifetime was calculated by inserting the Kaplan-Meier estimate into (9), and the bias was computed by *D*(*t*). For both the true and estimated loss of lifetime, the upper limit of the integrals in (9) was set to 40 years at which time the true survival was close to zero.

#### Results

**Fig. 4:**
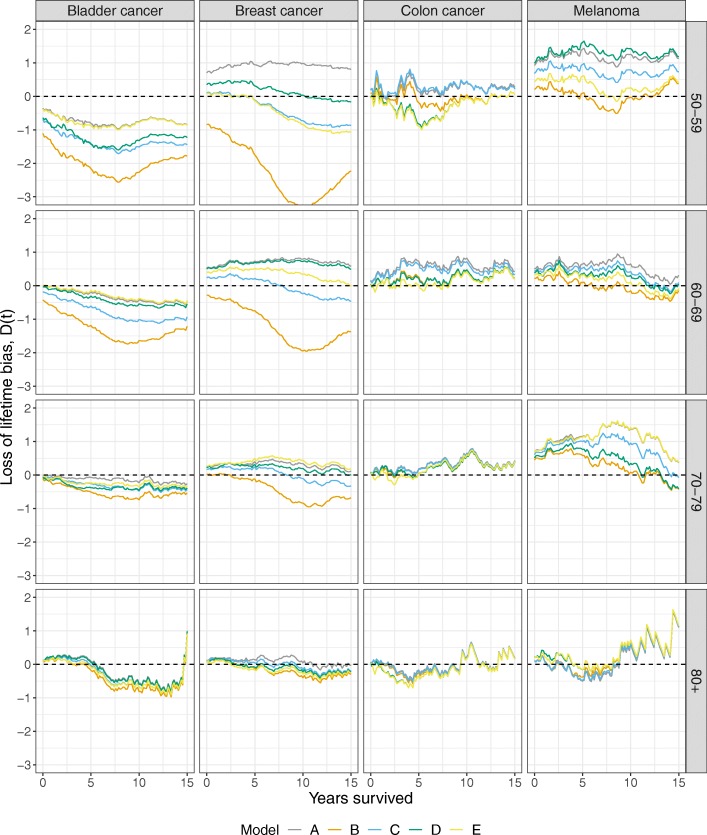
Time-varying loss of lifetime bias using the models in Table 1 for extrapolation in bladder cancer, breast cancer, colon cancer, and melanoma patients registered in the Danish Cancer registry

Figure 4 shows the bias function for each disease and each age group using the five models in Table 1. The corresponding survival curves can be found in Additional file 1: Figure S1-S4. The models displayed varying performance across the cancer types and age groups, but biases were commonly decreasing with increasing age. The extrapolation performance within bladder cancer was rather poor; in the age groups 50–59 and 60–69, the models consistently underestimated the loss of lifetime function with model B being the worst. Also for breast cancer, model B underestimated the loss of lifetime function while model C, which assumes statistical cure beyond the follow-up, provided improved results. In breast cancer, the two FMC models resulted in rather different loss of lifetime biases, but the bias was not consistently better in one model. For colon cancer where statistical cure is typically displayed, all models performed fairly well in all age groups and among the melanoma patients, model B had the best performance.

Overall, no model was consistently superior to the others, but in scenarios of statistical cure, there was a slight advantage of using cure models. However, in scenarios without statistical cure, models B and C were substantially biased.

### Analysis of Danish lymphoma registry data

#### Data description

To illustrate a potential clinical application of the proposed extrapolation techniques, we analyzed patient data from the Danish Lymphoma Registry, which covers 94.9% of all lymphoma cases in Denmark [21]. We included adult patients (≥18 years of age) diagnosed with diffuse large B-cell lymphoma (DLBCL, *n*=6639), follicular lymphoma (FL, *n*=3204), or mantle cell lymphoma (ML, *n*=980) in the period from 2000 to 2016. The follow-up period was terminated in June 2017 and the follow-up time was measured from time of diagnostic biopsy to death or censoring.

#### Population-based loss of lifetime

For each disease, three models were fitted, namely the NRS model with 6 knots, the ARS model with 7 knots, and the FMC model with 5 knots (corresponding to model A, B, and D in Table 1), resulting in the same number of parameters. Additional file 1: Figure S5 displays the relative survival of each disease and disease-specific summary measures are shown in Table 4.

**Table 4:** Median age, 5-year relative survival (RS), and loss of lifetime estimates at time zero in Danish diffuse large B-cell lymphoma (DLBCL), follicular lymphoma (FL), and mantle cell lymphoma (ML) patients

	Model	DLBCL	FL	ML
Median age (range)		68(18-101)	63(18-97)	70(28-99)
5-year RS (95% CI)	NRS	0.66(0.65-0.68)	0.9(0.88-0.91)	0.61(0.57-0.65)
	ARS	0.66(0.65-0.67)	0.9(0.88-0.91)	0.61(0.57-0.65)
	FMC	0.66(0.64-0.67)	0.9(0.88-0.91)	0.61(0.58-0.65)
Loss of lifetime (95% CI)	NRS	7.43(7.06-7.80)	4.58(3.73-5.42)	7.66(6.86-8.46)
	ARS	6.70(6.42-6.98)	3.57(3.13-4.02)	6.92(6.26-7.59)
	FMC	7.21(6.86-7.55)	3.97(3.24-4.70)	7.74(6.95-8.53)

**Fig. 5:**
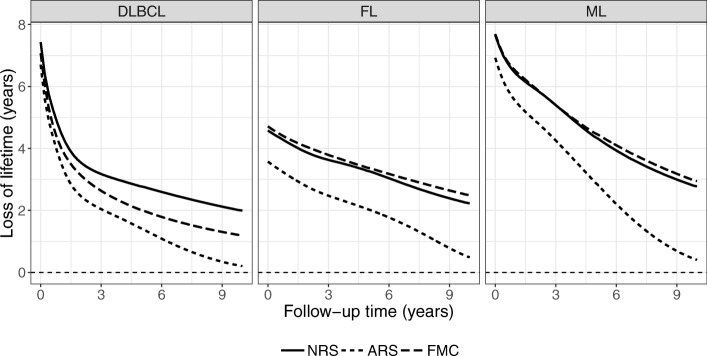
The loss of lifetime function in Danish diffuse large B-cell lymphoma (DLBCL), follicular lymphoma (FL), and mantle cell lymphoma (ML) patients

The estimated loss of lifetime function based on the three models is shown for each disease in Fig. 5. DLBCL and ML patients had a high loss of lifetime at diagnosis with a rapid decrease, while FL patients displayed a fairly low initial loss of lifetime with a slow improvement.

Clearly, the three models, despite being similar in the beginning of the follow-up, produce rather different conditional loss of lifetime estimates. At time zero, the maximal difference between the models is seen to be around 1 year for FL, for which the assumption of statistical cure is typically not reasonable. The model differences increased as time progressed, with the largest difference seen in ML patients. For DLBCL patients, the presented FMC model yielded a compromise between the NRS and ARS models which was seen by an intermediate loss of lifetime function. However, for the FL and ML patients where cure cannot usually be assumed, this model resembled the NRS model and even provided slightly higher loss of lifetime estimates.

#### Age dependent loss of lifetime

The patient age at diagnosis plays a crucial role for the individual expected residual lifetimes and thus also the loss of lifetime function. For the NRS model, a time-dependent age effect was specified, i.e.,

11 $$ R(t|a) = \exp{(-\exp{(s_{0}(x) + s_{a}(a)s_{1}(x)))}},  $$

where *a* is the patient age at diagnosis, *s*_*a*_(*a*) is a spline-based age effect and *s*_1_(*x*) is the corresponding time-effect. For the FMC model, (8), the same model was used for *S*_*u*_(*t*|***z***) and for *π*(***z***) an age dependent spline-based logistic model,

12 $$ \log\left(\frac{\pi}{1 - \pi}\right) = \beta_{0} + s_{a}(a),  $$

was chosen. Since none of the diseases showed a clear statistical cure trajectory, we did not consider the ARS model here. The number and location of the knots for the baseline spline function, *s*_0_(*x*), remained unchanged from “Population-based loss of lifetime” section. For *s*_*a*_(*a*), 4 knots placed at the 0%, 33%, 66%, and 100% quantiles of the patient age distribution were selected and the intercept was removed since this is already modelled by the baseline splines and *β*_0_. For *s*_1_(*x*), the number of knots was chosen to be 3 and 2 for the NRS model and the FMC model, respectively, yielding the same total number of parameters.

**Fig. 6:**
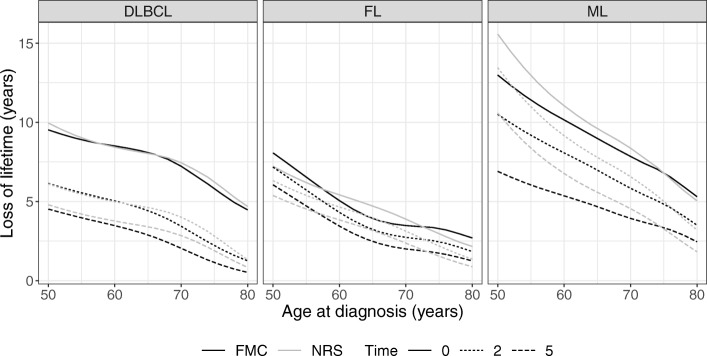
The loss of lifetime conditional on 0, 2, and 5 years of survival for female diffuse large B-cell lymphoma (DLBCL), follicular lymphoma (FL), and mantle cell lymphoma (ML) patients diagnosed in 2010 at varying ages

The loss of lifetime conditional on 0, 2, and 5 years of survival for female patients diagnosed in 2010 is shown in Fig. 6 for varying patient ages. In all three cancer types, the loss of lifetime decreased with increasing diagnostic age.

For DLBCL, the two models seemed to be in agreement across patient age. However, the agreement between the two models for 60–70 year old FL patients was poor, likely due to the different model assumptions. For ML, the model differences were larger for younger patients, likely due to the additional extrapolation needed to compute the loss of lifetime for these patients.

## Discussion

In (8) we introduced a novel model, which incorporates statistical cure by combining regular mixture cure models with spline-based survival models. This model was compared to the NRS model, which has a linear effect in the spline function after the last knot and the ARS model, which is constant after the last knot and thereby incorporates statistical cure. The simulations demonstrated a consistently good performance of the NRS model and the FMC model. The analysis of data from the Danish Cancer Registry did not show consistently satisfactory performance of any model, but in general assuming statistical cure at the end of the follow-up can lead to substantial biases in cases where this assumption is violated, while yielding good estimates when cure is reached. The NRS model performed slightly better than the FMC model in scenarios where statistical cure did not occur. This is likely due to the lack of identifiability often seen in cure models in cases where cure is not reached within the observed follow-up period [22], which ultimately may produce inaccurate extrapolations.

The present article expanded on the study of Andersson et al. [1] by evaluating the accuracy of the entire loss of lifetime function using three extrapolation approaches. While the loss of lifetime estimates at time zero in Fig. 4 seemed to be in agreement with the results reported by Andersson et al., where only 10 years of follow-up were used, the biases were not constant over time.

The general population survival probabilities for young patients are high and precise extrapolation of the relative survival is required to avoid a biased loss of lifetime function for these patients. Confirming this, we observed a higher bias among young patients which should be kept in mind when reporting loss of lifetime results. With longer follow-up and higher age, the bias will decrease and in future studies it would be of interest to estimate for a fixed age distribution, the amount of follow-up needed to provide sufficiently unbiased loss of lifetime estimates.

For some cancer types, the general population survival will likely not reflect the survival of the patients had they remained disease-free. The life style of patients diagnosed with, e.g., lung or skin cancer is likely different from the general population life style and hence the relative survival will not reflect the disease-specific (net) survival. However, this does not change the usability of the general population mortality rates to provide extrapolations of the survival function.

In contrast to net survival measures which are interpreted in the setting where the patient can only die from the disease of interest, the loss of lifetime measure provides a crude measure of the cancer-related mortality. In net measures, such as relative survival, it is often seen that elderly patients have an increased mortality since deaths from other causes are not taken into account. For young patients, even a small excess mortality may have a large impact on the loss of lifetime function as the expected lifetime without cancer is long. Therefore, it is often seen that young patients have a higher loss of lifetime than elderly patients.

An alternative to the unrestricted loss of lifetime, where extrapolation is avoided, can be obtained by replacing the upper limit of the integrals in (9) by a fixed time point *τ*. In this setting, pseudo-values and flexible parametric survival models have previously been recommended for computing the mean survival time [23] and could also be used for estimating the loss of lifetime function. Using the three models to estimate the restricted loss of lifetime would likely yield fairly similar estimates due to the model similarities in the first part of the follow-up (Additional file 1: Figure S5). However, interpretation of the restricted loss of lifetime is not straightforward and the measure does not capture the full disease burden.

## Conclusion

Since there is no way of assessing the performance of extrapolations applied to data with limited follow-up, the inconsistencies between the simulation results and the full follow-up data analysis emphasize the need for sensitivity analyses.

We therefore recommend that extensive sensitivity analyses are performed both with respect to the assumptions of the relative survival model as well as the number and location of the knots of the splines as recommended previously [10, 13].

## Additional file

Additional file 1 Supplementary figures. Description of data: Figure S1-S4 displays the extrapolated overall survival for the four cancer types considered in the analysis of data from the Danish Cancer Registry. Figure S5 displays the relative survival of the three lymphoma types considered in “Population-based loss of lifetime” section. (DOCX 248 kb)

## Abbreviations

ARS: Andersson et al. [13] relative survival
DLBCL: Diffuse large B-cell lymphoma
FL: Follicular lymphoma
FMC: Flexible mixture cure
ML: Mantle cell lymphoma
NRS: Nelson et al. [10] relative survival
