# Supplementary material for: Estimating the loss of lifetime function using flexible parametric relative survival models
Source: BMC Med Res Methodol. 2019 Jan 28;19:23. doi: 10.1186/s12874-019-0661-8 (PMC6350283; doi:10.1186/s12874-019-0661-8)
Supplement: Supplementary file 1 — Supplementary figures. Description of data: Figure S1-S4 displays the extrapolated overall survival for the four cancer types considered in the analysis of data from the Danish Cancer Registry. Figure S5 displays the relative survival of the three lymphoma types considered in “Population-based loss of lifetime” section. (DOCX 248 kb) [file 12874_2019_661_MOESM1_ESM.docx]

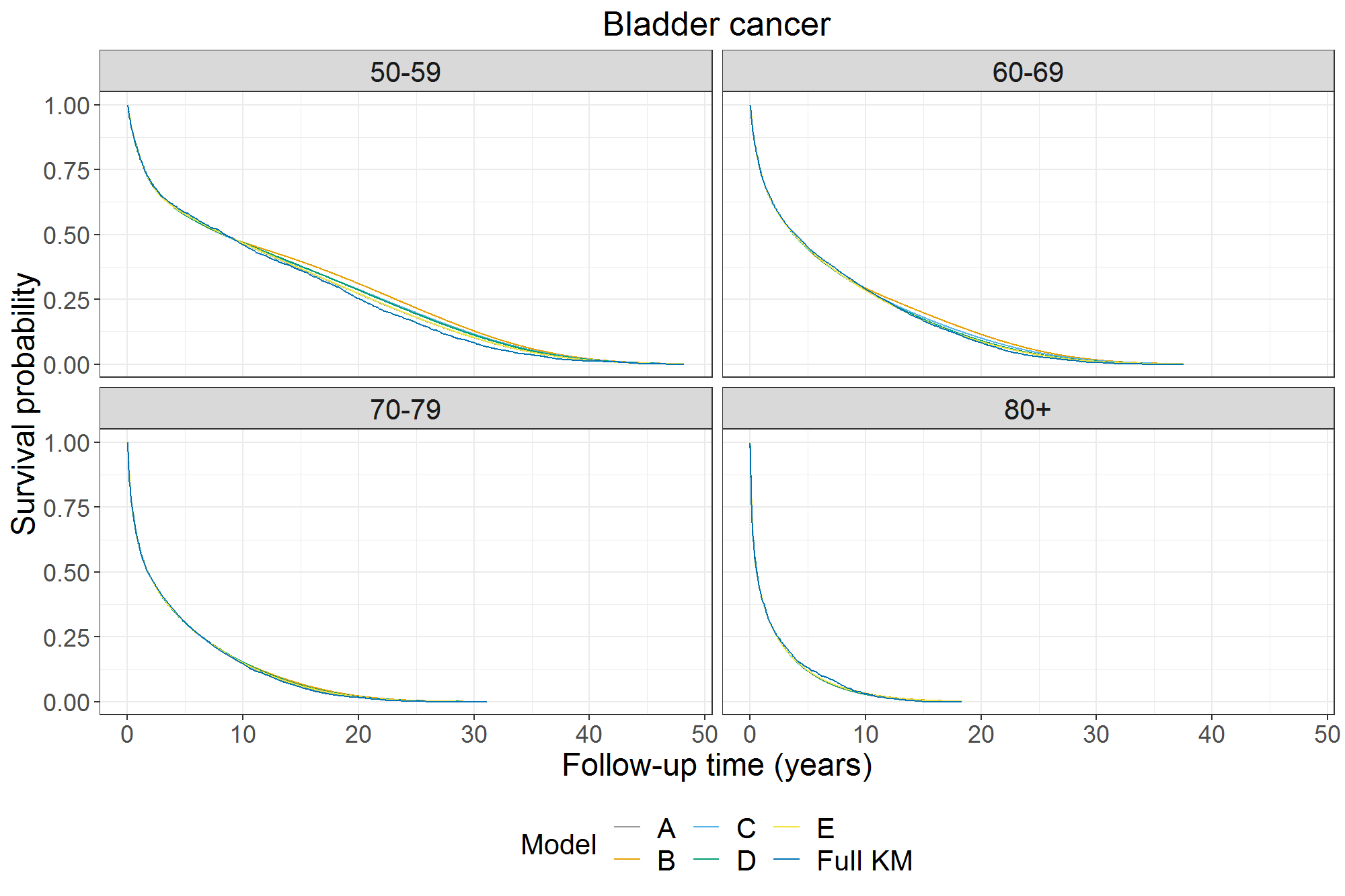
Figure S1: The extrapolated survival function of bladder cancer patients based on 5 relative survival models.


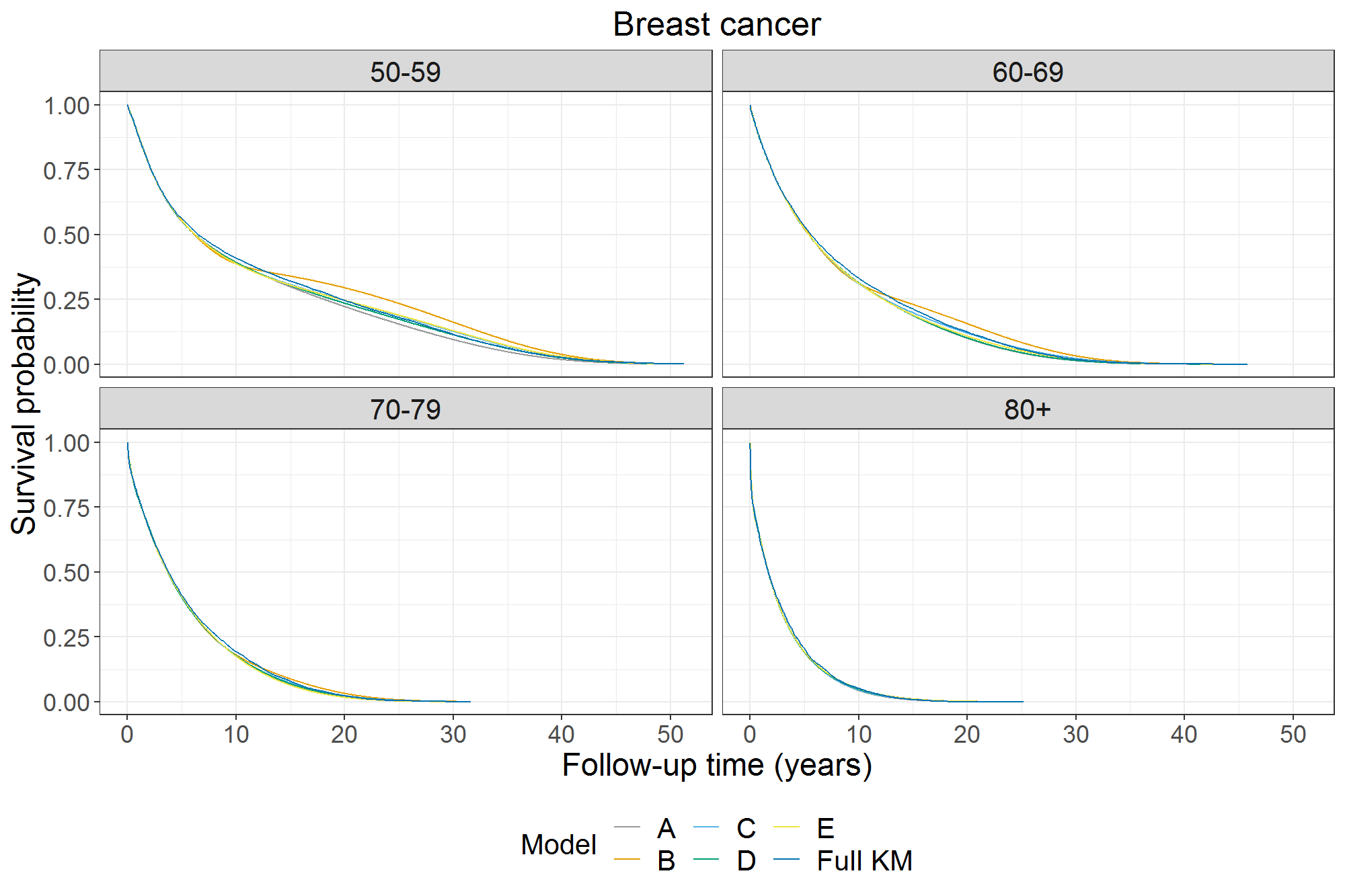
Figure S2: The extrapolated survival function of breast cancer patients based on 5 relative survival models.


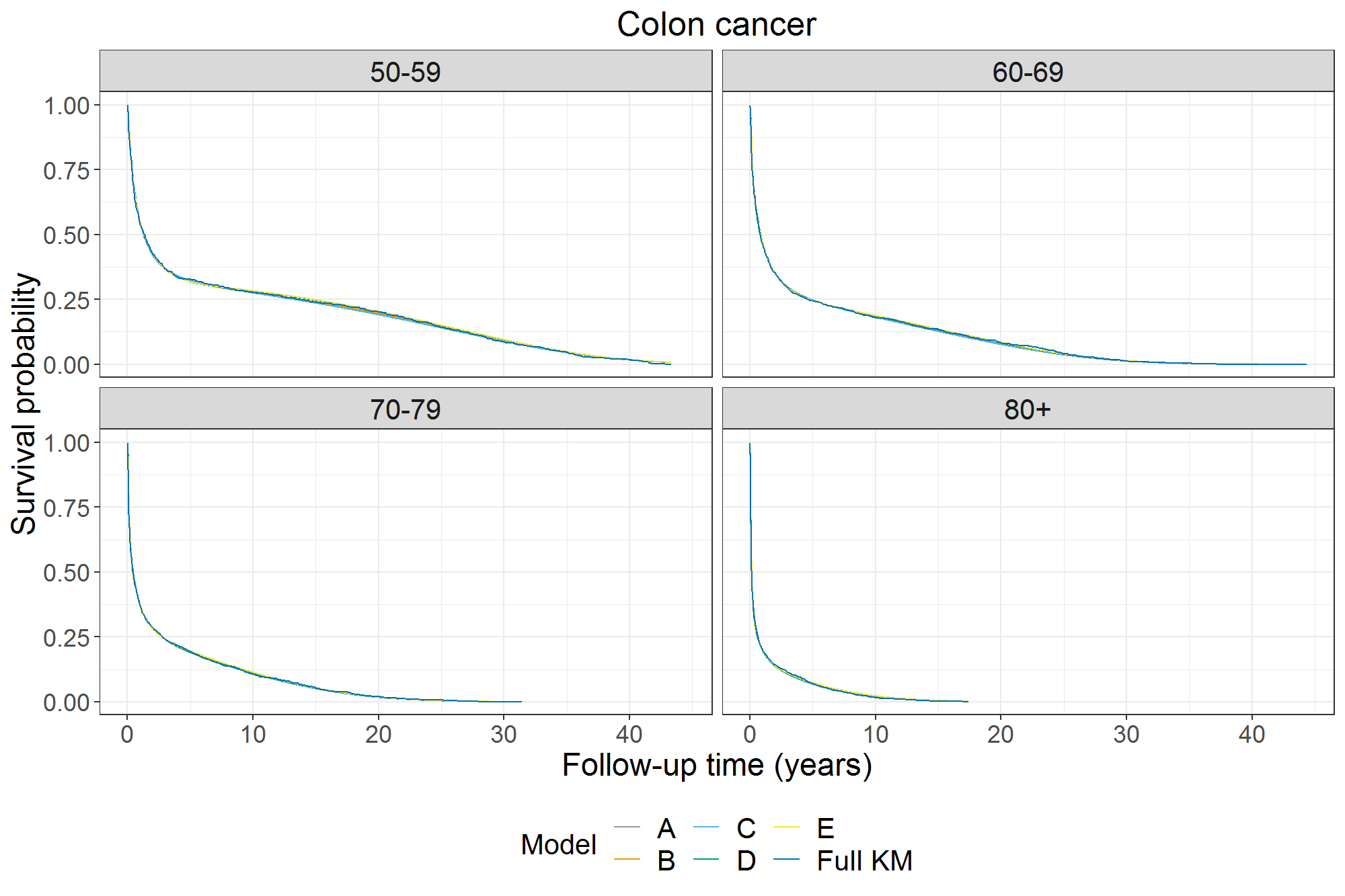
 Figure S3: The extrapolated survival function of colon cancer patients based on 5 relative survival models.


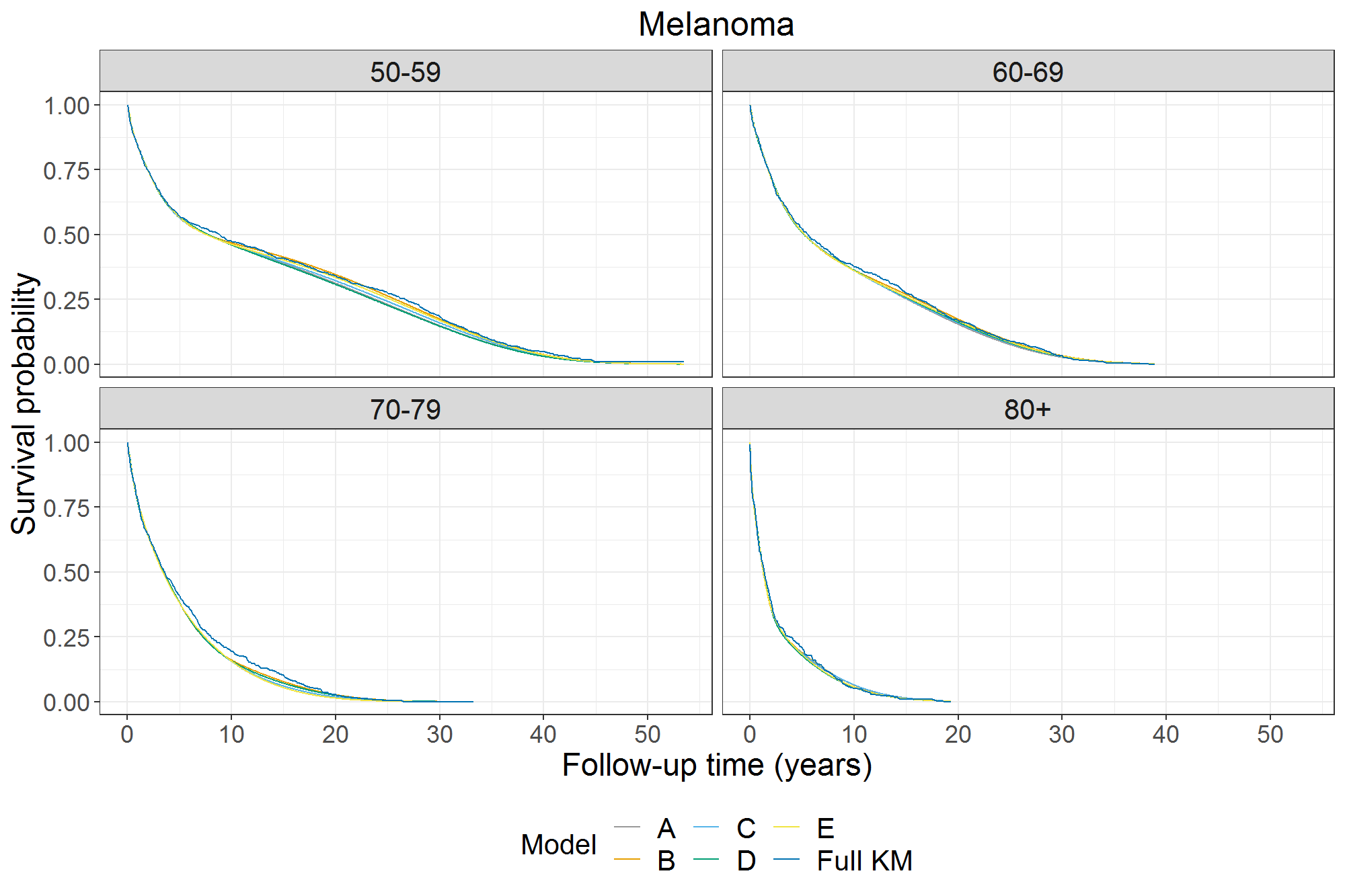
Figure S4: The extrapolated survival function of melanoma patients based on 5 relative survival models.


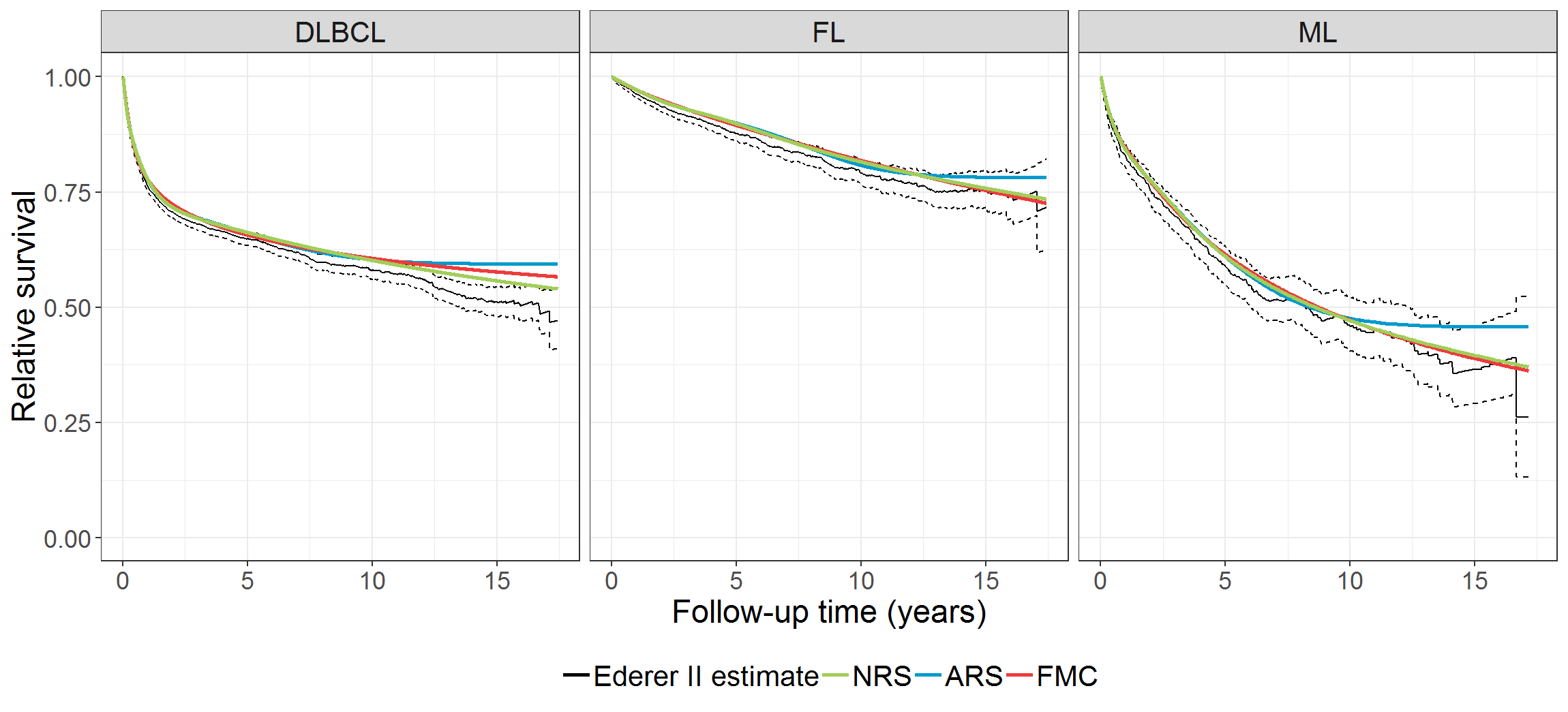
Figure S5: The relative survival of Danish diffuse large B-cell lymphoma (DLBCL), follicular lymphoma (FL), and mantle cell lymphoma (ML) patients calculated by the Ederer II method including confidence intervals (dashed lines), the NRS model, the ARS model, and the FMC model.
